# Cdc42 activation couples fluid shear stress to apical endocytosis in proximal tubule cells

**DOI:** 10.14814/phy2.13460

**Published:** 2017-10-16

**Authors:** Sohinee Bhattacharyya, Frédéric G. Jean‐Alphonse, Venkatesan Raghavan, Jennifer C. McGarvey, Youssef Rbaibi, Jean‐Pierre Vilardaga, Marcelo D. Carattino, Ora A. Weisz

**Affiliations:** ^1^ Renal‐Electrolyte Division Department of Medicine University of Pittsburgh School of Medicine Pittsburgh Pennsylvania; ^2^ Laboratory for GPCR Biology Department of Pharmacology and Chemical Biology University of Pittsburgh School of Medicine Pittsburgh Pennsylvania; ^3^ Present address: Cell, Developmental and Cancer Biology, Oregon Health & Science University Portland OR; ^4^ Present address: QuintilesIMS New York New York; ^5^ Present address: Heptares Therapeutics Welwyn Garden City Hertfordshire United Kingdom

**Keywords:** Calcium, calmodulin, cilia, cubilin, endocytosis, fluid shear stress, megalin, proximal tubule

## Abstract

Cells lining the kidney proximal tubule (PT) respond to acute changes in glomerular filtration rate and the accompanying fluid shear stress (FSS) to regulate reabsorption of ions, glucose, and other filtered molecules and maintain glomerulotubular balance. Recently, we discovered that exposure of PT cells to FSS also stimulates an increase in apical endocytic capacity (Raghavan et al. PNAS, 111:8506–8511, 2014). We found that FSS triggered an increase in intracellular Ca^2+^ concentration ([Ca^2+^]_i_) that required release of extracellular ATP and the presence of primary cilia. In this study, we elucidate steps downstream of the increase in [Ca^2+^]_i_ that link FSS‐induced calcium increase to increased apical endocytic capacity. Using an intramolecular FRET probe, we show that activation of Cdc42 is a necessary step in the FSS‐stimulated apical endocytosis cascade. Cdc42 activation requires the primary cilia and the FSS‐mediated increase in [Ca^2+^]_i_. Moreover, Cdc42 activity and FSS‐stimulated endocytosis are coordinately modulated by activators and inhibitors of calmodulin. Together, these data suggest a mechanism by which PT cell exposure to FSS is translated into enhanced endocytic uptake of filtered molecules.

## Introduction

Renal epithelial cells that line the kidney nephron encounter wide fluctuations in the magnitude of FSS due to frequent changes in glomerular filtration rate (GFR). FSS produced by the movement of the glomerular ultrafiltrate through the renal tubule acutely regulates PT ion transport. This mechanism prevents loss of solute after increases in GFR and maintains the adequate distal delivery of sodium and fluid when GFR is reduced. PT cells respond to mechanical forces, including FSS, with immediate downstream signal transduction events as well as long‐term adaptation mechanisms that enable them to fine‐tune their cytoskeleton structure, intercellular junctions, and cell‐substratum interactions to adjust to the mechanical challenge (Weinbaum et al. 2010; Raghavan and Weisz 2015).

High tubular flow rates are common complications of kidney diseases, and FSS is instrumental in coordinating biochemical signaling pathways that regulate cell morphology, migration and growth (Rohatgi and Flores 2010). It is well recognized that the primary cilia present on renal epithelial cells acts as a flow sensor that couples upstream flow stimuli to rapid increases in [Ca^2+^]_i_ (Praetorius 2015). Mutations in ciliary proteins are associated with deregulated FSS‐mediated [Ca^2+^]_i_ responses and with the development of polycystic kidney disease, suggesting that failure to properly sense biomechanical signals may contribute to cyst development (Yoder 2007; Kotsis et al. 2013). However, despite considerable evidence of the crucial role of FSS in regulating renal epithelial integrity and function, the molecular mechanisms by which FSS is transduced into downstream signals remain unclear (Delling et al. 2016).

An essential function of the PT is the clathrin‐dependent apical uptake of filtered low molecular weight proteins and albumin by the multiligand receptors megalin and cubilin (Eshbach and Weisz 2017). We previously reported that exposure of kidney PT cells to FSS causes a rapid and sustained increase in endocytic capacity (Raghavan et al. 2014). FSS also triggered an increase in [Ca^2+^]_i_ in these cells that required the presence of primary cilia and extracellular release of ATP. Our findings led us to hypothesize that flow‐mediated Ca^2+^ signaling initiates a downstream cascade that coordinates reorganization of the cytoskeleton to stimulate endocytosis. However, how increased [Ca^2+^]_i_ ultimately stimulates endocytic uptake in PT cells is not known.

Cdc42 is a member of the Rho family of GTPases that function to regulate numerous cellular activities, including cytoskeletal dynamics and endocytosis. Cdc42 is known to be activated in response to increased [Ca^2+^]_i_ and has been implicated in endothelial cell responses to FSS (Li et al. 1999; Tzima et al. 2003). The subapical domain of PT cells is actin‐rich and we previously hypothesized that changes in actin dynamics might regulate apical endocytosis in response to FSS (Raghavan et al. 2014). In this study, we use two complementary approaches to investigate the underlying intracellular signaling pathway that couples FSS to apical endocytosis. In the first, we used sensitive fluorescence resonance energy transfer (FRET) probes to monitor activation of Cdc42 in PT cells upon exposure to laminar FSS or pharmacologic modulators of [Ca^2+^]_i_‐dependent signaling pathways. In a parallel approach, we measured the effects of these maneuvers on apical endocytosis. Our data suggest a mechanism by which Cdc42 alters actin dynamics to enhance PT endocytic uptake in response to FSS.

## Materials and Methods

### Cell Culture

Opossum kidney OK cells (*Didelphis virginiana,* adult female, kidney cortex) were originally provided by Moshe Levi (University of Colorado) and cultured in DMEM/F12 Hamm with 10% FBS. For most experiments, 4.5 × 10^5^ cells were plated in Ibidi *μ*‐slide I 0.4 or 0.8 collagen‐coated chambers (Ibidi). For confocal imaging studies, 3 × 10^5^ cells/well were plated in Ibidi *μ*‐slide VI 0.4 chambers. Medium was replaced twice daily for 3 days before experiments. For FRET and endocytosis experiments, cells were pretreated with drugs [ML141 (Tocris‐4266, 10 *μ*mol/L); Calp3 (Tocris‐2321, 5 *μ*mol/L; ryanodine (Tocris‐1329, 25 *μ*mol/L), or W13 (Tocris‐0361, 60 *μ*mol/L), all prepared at ≥1000× stocks in DMSO or water as recommended] for 1 h in culture medium prior to initiation of FSS. Cells were transfected with Raichu plasmids immediately prior to plating using Lipofectamine 3000 reagent (Invitrogen) according to manufacturer guidelines. Where indicated, cells were deciliated by incubation for 3 h in 30 mmol/L ammonium sulfate in FBS‐supplemented medium as previously described (Raghavan et al. 2014) and allowed to recover overnight where indicated.

### Measurement of Cdc42 activation using FRET

The Raichu‐Cdc42 intramolecular FRET probe has been described previously (Itoh et al. 2002; Nakamura et al. 2006). In brief, Raichu‐Cdc42 consists of YFP, the CRIB domain of the Cdc42 effector protein PAK1, Cdc42, CFP, and the CAAX box of *K*_*i*_‐Ras to enable membrane tethering, all linked via spacer sequences. The probe is designed so that the signal‐induced conformational change in the probe brings CFP into close proximity with YFP, which results in a decrease in CFP fluorescence and a concomitant increase in YFP fluorescence.

Cells expressing the Raichu‐Cdc42 probe were imaged using a Nikon Ti‐E microscope (Nikon) equipped with a Z‐driven piezo motor. Images were acquired using the Nikon A1 confocal unit through a 60 × 1.45 N/A objective (Nikon). Data were acquired using Nikon Elements Software (Nikon Corporation). Fluorescent proteins were excited with 440‐nm (CFP) and 514‐nm (YFP) lasers (Melles Griot). Six to twenty cells [regions of interest (ROIs)] that had comparable starting ratios of YFP/CFP were selected prior to initiating the experiment. For FRET, an excitation wavelength of 440 ± 10 nm was used to excite CFP, and YFP and CFP emissions were collected using 535 ± 12 nm (YFP) and 480 ± 15 nm (CFP) filters. A syringe pump was connected to the cell‐seeded Ibidi *μ*‐slide I 0.4 microchannel slides and a flow rate of 111 *μ*L/min was used to generate unidirectional laminar shear stress of ~0.1 dyne/cm^2^ according to the manufacturer's instructions. Typically, the experimental timeline was as follows: baseline images (static condition images) were acquired every minute for 10 min, after which the cells were exposed to FSS for 30 min. Subsequently, the flow was stopped and the cells were imaged for an additional 20 min under static conditions. NIS software was used to determine the fluorescence intensity for each ROI as well as the background. Further analysis was performed with Excel software (Microsoft). YFP divided by CFP fluorescence ratios were obtained by taking the fluorescence ratio of background‐normalized YFP and background‐normalized CFP signal intensities. These values were normalized against basal signal intensities (before initiation of flow). The average and standard deviation were calculated from the YFP/CFP ratios for at least three independent experiments for each condition and statistical significance assessed by two‐way ANOVA.

### Quantitation of endocytic uptake in response to FSS

OK cells cultured in Ibidi *μ*‐slide I 0.4 chambers were exposed to 0.1 dyne/cm^2^ laminar FSS for 1 h at 37°C in the presence of 40 *μ*g/mL AlexaFluor 647‐albumin (Molecular Probes) in DMEM/F12 Hamm medium supplemented with 25 mmol/L HEPES, pH 7.5, then washed five times with ice cold PBS supplemented with 1 mmol/L Ca^2+^ and 1 mmol/L Mg^2+^ (PBS‐CM). Cells were solubilized in 200 *μ*L 50 mmol/L Tris‐HCl, 1% (v/v) IGEPAL, 0.4% deoxycholate, 62.5 mmol/L EDTA, pH 8.0 at ambient temperature for 30 min and fluorescence quantified using a Glomax‐Multi Detection spectrofluorimeter (Promega). Background values (lysate buffer) were <10% of the lowest experimental values and were subtracted prior to data analysis.

### Confocal imaging of albumin uptake

For experiments measuring effects of fluid shear stress (FSS) on endocytosis, cells in Ibidi *μ*‐slide VI 0.4 six‐well chambers were perfused or maintained under static conditions for 1 h at 37°C. After washing, cells were fixed with 4% paraformaldehyde for 20 min at ambient temperature, rinsed twice with PBS‐CM, and 50 *μ*L of ProlongGold (Invitrogen) added to each well. The chambers were imaged using a Leica TCS SP5 confocal microscope and maximum intensity projections of stacks were processed identically using Fiji and Adobe Photoshop.

### [Ca^2+^]_i_ measurements

OK cells cultured on collagen‐coated Ibidi *μ*‐slide I (0.8‐mm clearance) chambers were loaded with Fura‐2 AM (2.5 *μ*mol/L) in HEPES‐buffered saline (HBS: 135 mmol/L NaCl, 4.5 mmol/L KCl, 2.5 mmol/L CaCl_2_, 1 mmol/L MgCl_2_, 10 mmol/L HEPES, 10 mmol/L glucose, pH 7.4) for 20 min at 37°C, rinsed with HBS, and incubated for 10 min to de‐esterify the dye at ambient temperature. Ibidi *μ*‐slides with Fura‐2 loaded cells were placed in a stage top incubator (Tokai Hit) set at 37°C for 10 min. Cells were imaged with a Nikon Eclipse Ti upright live cell microscope using an ORCA‐Flash 2.8 camera (Hamamatsu). Cells were excited with a PhotoFluor II metal halide light source (89 North) connected to a Lambda 10‐3 filter wheel system (Sutter Instruments) with 340‐ and 380‐nm filters (Chroma Technology). To generate a FSS of 0.1 dyne/cm^2^, Ibidi *μ*‐slides were perfused with an infusion pump (Harvard Apparatus) at a flow rate of 400 *μ*L/min. Temperature was maintained at 37°C throughout the experiment using an inline heater (SH27B, Warner Instruments) controlled by a dual channel bipolar temperature controller (TC‐344B, Warner Instruments).

Baseline measurements were acquired every 10 sec for 1 min before initiation of FSS and then every 5 sec, 10 sec, or 30 sec. In situ calibration with ionomycin (Alomone) was conducted to define the Ca^2+^ calibration parameters under zero and saturating [Ca^2+^]_i_ concentrations. Cells were perfused with Ca^2+^‐free buffer (135 mmol/L NaCl, 4.5 mmol/L KCl, 2 mmol/L EGTA, 1 mmol/L MgCl_2_, 10 mmol/L HEPES, 10 mmol/L glucose, pH 7.4) containing 2.5 *μ*mol/L ionomycin to estimate ratios at zero concentration of [Ca^2+^]_i_. Subsequently, cells were perfused with HBS with 2.5 *μ*mol/L ionomycin to estimate Fura‐2 ratios at saturating levels of [Ca^2+^]_i_. NIS Elements (Nikon) was used to select individual cells (6–10 per experiment) and the ratio of 340:380‐nm emission intensity was calculated at each time point. The [Ca^2+^]_i_ values of cells at each time point was normalized to the initial [Ca^2+^]_i_ value (at *t* = 0 sec) to estimate the fold changes in [Ca^2+^]_i_ over time. [Ca^2+^]_i_ changes across experimental and control conditions were averaged across 4–6 experiments (34–43 cells per condition).

## Results

### Cdc42 is activated by FSS and is required for the endocytic response to FSS

To determine whether exposure to FSS activates Cdc42, we transfected OK cells with the “Ras and interacting protein chimeric unit” Raichu‐Cdc42 construct and measured time courses of intramolecular FRET changes as readout for activation of this small GTPase in live cells (Itoh et al. 2002). Activation of Cdc42 in this membrane‐tethered construct facilitates binding of the attached CRIB domain of the Cdc42 effector PAK1. This change brings CFP into close proximity with YFP, causing a decrease in CFP fluorescence and a concomitant increase in YFP fluorescence due to FRET. The high affinity of the intramolecular PAK1 interaction with Cdc42 in the Raichu‐Cdc42 construct is essentially irreversible upon activation of Cdc42 (Thompson et al. 1998; Itoh et al. 2002). Despite this limitation, this probe is a useful tool to confirm Cdc42 activation in response to acute stimuli.

**Figure 1:**
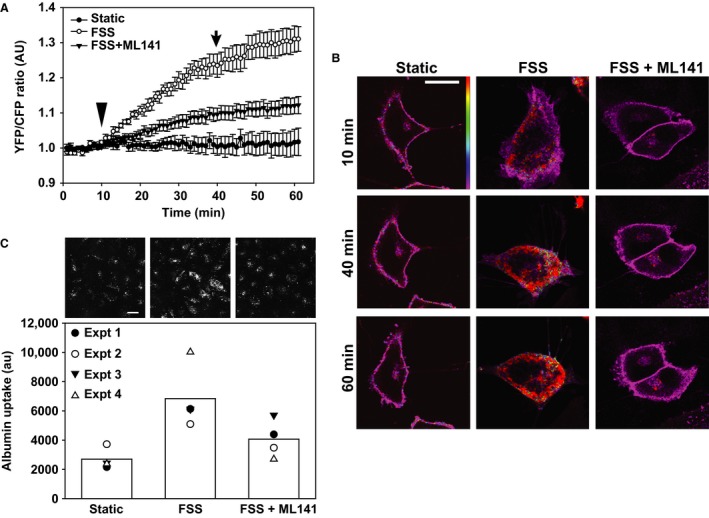
Cdc42 is activated by fluid shear stress and is required for the endocytic response to flow. (A) FRET measurements of OK cells transfected with the Raichu‐Cdc42 FRET probe and cultured in Ibidi chambers. Cells were maintained under static conditions throughout the 1 h imaging period or were exposed to 0.1 dyne/cm^2^ FSS starting at 10 min (arrowhead) until 40 min (arrow). The Cdc42 inhibitor ML141 (10 *μ*mol/L) was included where indicated. Data (mean ± SEM) from five independent experiments (6–20 regions of interest analyzed per experiment) are plotted. All curves are significantly different from each other (*P* < 0.001) by two‐way ANOVA. (B) YFP/CFP merged images of Raichu‐Cdc42 taken at the indicated times reveal selective activation of Cdc42 in cells exposed to FSS. Note the increase in protrusions and change in cell shape upon exposure to FSS in control but not ML141‐treated cells. Scale bar: 25 *μ*mol/L. (C) OK cells cultured on Ibidi chambers were incubated for 1 h at 37°C with AlexaFluor 647‐albumin under static conditions or at 0.1 dyne/cm^2^ FSS, and cell associated albumin quantified as described in Methods. ML141 was included where indicated. Data from four individual experiments, each shown using a different symbol, are plotted, and the bar shows the mean uptake for each condition. Representative images from a separate experiment in which cells were fixed and albumin uptake imaged using confocal microscopy are shown above each bar. Scale bar: 25 *μ*m.

FRET ratios (YFP/CFP) were stable (or sometimes decreased slightly due to unequal bleaching of CFP and YFP) when cells were maintained under static conditions over a 1 h period (Fig. 1A and B). In contrast, when cells were imaged for 10 min and then exposed to 0.1 dyne/cm^2^ FSS (arrowhead in Fig. 1A), Cdc42 became gradually activated. Moreover, cells exposed to FSS developed protrusions, changed shape, and migrated more rapidly compared with cells maintained under static conditions, consistent with known effects of activated Cdc42 on cellular dynamics (Fig. 1B). The FRET signal remained steady or even increased slightly even after FSS was stopped (arrow in Fig. 1A), consistent with the irreversibility of this FRET probe.

ML141 is a high affinity and selective inhibitor of Cdc42 [EC_50_~1.4 *μ*mol/L (Hong et al. 2013)] that exhibits minimal inhibition of other related GTPases including Rac or Rho at concentrations up to 100 *μ*mol/L (Surviladze et al. 2010). Incubation of cells with 10 *μ*mol/L ML141 (Surviladze et al. 2010; Hong et al. 2013), starting 1 h prior to the experiment, reduced the FSS‐induced increase in Cdc42 FRET, and also prevented morphological and migratory changes in cells (Fig. 1A and B).

To test whether Cdc42 activation is necessary for the stimulation of endocytosis that we previously observed in response to FSS, we incubated OK cells under static conditions or at 0.1 dyne/cm^2^ for 1 h in the presence of 40 *μ*g/mL AlexaFluor 647‐albumin, then washed and solubilized the cells and quantified cell‐associated fluorescence using spectrofluorimetry (Fig. 1C, lower panel). Exposure to acute FSS increased albumin uptake in cells by ~50–100%. Parallel studies in which cells were treated similarly, then fixed and imaged by confocal microscopy, confirmed an increase in intracellular albumin in cell exposed to FSS compared with cells maintained under static conditions (Fig. 1C, upper panel). This increase was markedly inhibited when cells were preincubated with ML141 for 1 h prior to addition of AlexaFluor 647‐albumin (Fig. 1C). Consistent with the well‐established role of Cdc42 in modulating actin dynamics, we confirmed that endocytosis in OK cells is actin dependent, as albumin uptake was profoundly inhibited by cytochalasin D (S.B. and V.R., unpublished observations). We conclude that activation of Cdc42 is an important step in the cascade leading from FSS to increased endocytosis.

### Cilia and calcium are required for Cdc42 activation by FSS

**Figure 2:**
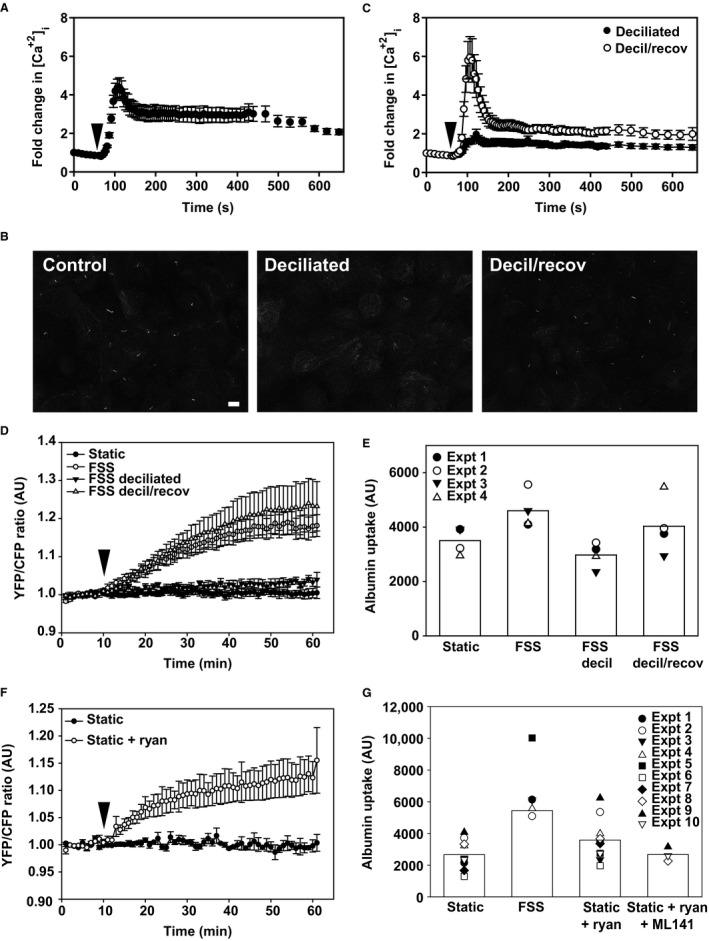
Primary cilia and calcium are required for the activation of Cdc42 by FSS. (A) OK cells cultured in Ibidi chambers were loaded with Fura‐2 and [Ca^2+^]_i_ levels were monitored before and upon exposure to 0.1 dyne/cm^2^ FSS (arrowhead). (B) Indirect immunofluorescence of acetylated tubulin staining to visualize primary cilia in control cells, cells deciliated with ammonium sulfate (decil), and deciliated cells after overnight recovery to enable cilia regrowth (decil/recov). Scale bar: 10 *μ*m. (C) [Ca^2+^]_i_ levels were monitored as above in deciliated cells immediately after deciliation and after overnight recovery. Kruskal–Wallis test (nonparametric) followed by Dunn's multiple comparisons test confirmed statistically significant differences in peak [Ca^2+^]_i_ levels between control and deciliated cells (*P* < 0.001) and between deciliated and recovered cells (*P* < 0.001). (D) Cdc42 is not activated in deciliated cells exposed to FSS. Cells were deciliated for 3 h prior to exposure to 0.1 dyne/cm^2^ FSS for 30 min starting at 10 min (arrowhead) or incubated overnight to recover cilia prior to measuring the Raichu‐Cdc42 FRET response to FSS. Mean ± SEM is plotted (static, *n* = 6; FSS,* n* = 7; deciliation, *n* = 8, decil/recov, *n* = 3). Profiles for FSS versus rescue and static versus deciliated conditions were not significantly different from each other; *P* values for all other comparisons were <0.001 by two‐way ANOVA. (E) Endocytosis is not stimulated by FSS in deciliated cells. OK cells cultured on Ibidi chambers were deciliated immediately prior to quantitation of AlexaFluor 647‐albumin uptake or incubated overnight to recover cilia. Data from four individual experiments, each shown using a different symbol, are plotted, and the bar shows the mean uptake for each condition. Representative images from a separate experiment in which cells were fixed and albumin uptake imaged using confocal microscopy are shown above each bar. Scale bar: 25 *μ*m. (F) Ryanodine activates Cdc42 in the absence of FSS. FRET ratios were monitored in OK cells transfected with the Raichu‐Cdc42 under static conditions. Ryanodine (25 *μ*mol/L) was added at 10 min (arrowhead) and the incubation continued for an additional 50 min. The mean of from three experiments is plotted. Profiles for static versus static + ryanodine are significantly different from each other (*P* < 0.001) by two‐way ANOVA. (G) Ryanodine‐stimulated albumin uptake in the absence of FSS is inhibited by ML‐141. Data from 10 experiments are plotted, and the bar shows the mean uptake for each condition.

Estimates of FSS experienced by cells lining the PT differ widely, from ~0.1 to ≥1 dyne/cm^2^ (Essig and Friedlander 2003; Du et al. 2004; Duan et al. 2008; Jang et al. 2013). We previously demonstrated that exposure of OK cells to ≥2 dyne/cm^2^ FSS causes a transient spike in [Ca^2+^]_i_ that requires the primary cilium, extracellular Ca^2+^, and Ca^2+^ release from intracellular stores (Raghavan et al. 2014). Because Ca^2+^ signaling may vary with FSS (Xu et al. 2009; Liu et al. 2011), we modified our imaging setup to enable us to monitor changes in [Ca^2+^]_i_ at the lower FSS used in our Cdc42 activation and endocytosis experiments. OK cells were loaded with Fura‐2 and imaged prior to and during exposure to 0.1 dyne/cm^2^ FSS. Initial [Ca^2+^]_i_ concentrations were somewhat variable when measured using Fura‐2, however, fold‐dependent increases in [Ca^2+^]_i_ were very consistent across cells, similar to our previous observations (Raghavan et al. 2014). As shown in Figure 2A, exposure to acute FSS caused a transient 4–5‐fold increase in [Ca^2+^]_i_ that declined within ~2 min to a new higher baseline (~3‐fold higher than starting levels) and remained stable for at least 10 min. This profile is similar to that observed upon stimulation of Ca^2+^ signaling in vivo and in studies in MDCK cells using FSS <1 dyne/cm^2^ (Rydholm et al. 2010; Szebenyi et al. 2015). In contrast, in previous studies where OK and other cells were exposed to higher shear stress, [Ca^2+^]_i_ levels rapidly increased 2–4‐fold but rapidly (<5 min) returned to near baseline levels (Nauli et al. 2003; Raghavan et al. 2014).

We previously demonstrated that deciliation of OK cells compromised the Ca response to ≥2 dyne/cm^2^ FSS. To determine whether this also occurs at 0.1 dyne/cm^2^, we incubated cells with 30 mmol/L ammonium sulfate for 3 h to remove cilia, and loaded them with Fura‐2 immediately afterward or after overnight incubation to recover cilia (Raghavan et al. 2014). Indirect immunofluorescence of cells with antiacetylated tubulin to visualize primary cilia confirmed that ammonium sulfate treatment effectively removed primary cilia, and that deciliated cells regenerated primary cilia after overnight incubation (Fig. 2B). Deciliated cells loaded readily with Fura‐2, suggesting that membrane permeability was not compromised by this maneuver. Similar to our previous results, the [Ca^2+^]_i_ response to FSS was markedly blunted in deciliated cells (Fig. 2C). When cells were allowed to recover overnight after deciliation the response to FSS was similar to that in control cells (Fig. 2C).

Next, we monitored FRET in cells expressing Raichu‐Cdc42 to determine whether intact primary cilia are required for FSS‐dependent activation of Cdc42. No increase in FRET was observed in deciliated cells upon exposure to FSS, consistent with a requirement for ciliary‐mediated increase in [Ca^2+^]_i_ to activate Cdc42 (Fig. 2D). FSS‐dependent activation of Cdc42 was fully restored when cells were allowed to recover overnight (Fig. 2D). Additionally, we confirmed our previously published observation that deciliation impairs albumin uptake in response to FSS, and that this response is restored after cell recovery [(Fig. 2E) and (Raghavan et al. 2014)].

To assess whether the increase in [Ca^2+^]_i_ upon exposure to FSS is upstream of Cdc42 activation, we asked whether increasing [Ca^2+^]_i_ by addition of ryanodine is sufficient to activate Cdc42 in the absence of FSS. As shown in Figure 2F, addition of ryanodine increased Cdc42 FRET, albeit to a lesser degree compared with FSS. Similarly, ryanodine enhanced endocytic uptake of albumin in OK cells maintained under static conditions (Fig. 2G). Importantly, the ryanodine‐mediated increase in albumin uptake was not observed in the presence of ML141 (Fig. 2G). Together, these data suggest that the primary cilium is required for FSS‐dependent mobilization of [Ca^2+^]_i_ that activates Cdc42 and stimulates FSS‐dependent endocytosis.

### CaM and CaMKII mediate the activation of Cdc42 and the endocytic response to FSS

**Figure 3:**
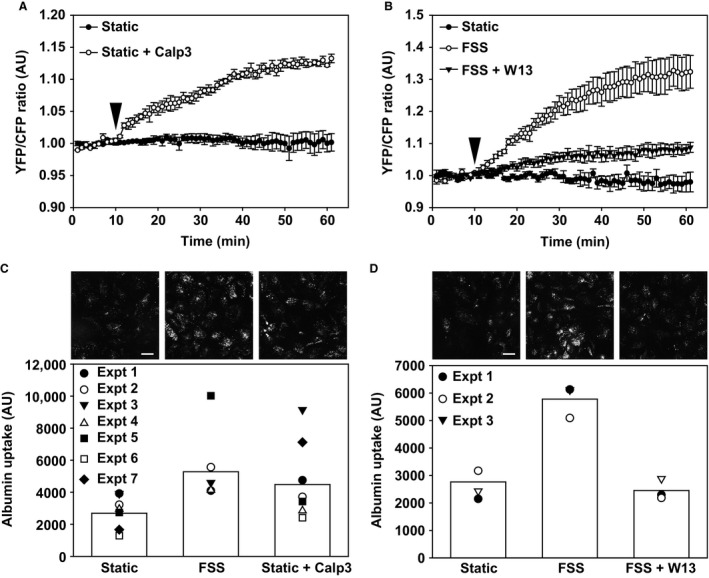
CaM activation is required for Cdc42 activation and FSS‐stimulated endocytosis. (A) FRET ratios were monitored in OK cells transfected with the Raichu‐Cdc42 under static conditions. The calmodulin activator Calp3 (5 *μ*mol/L) was added at 10 min (arrowhead). The mean ± SEM of three independent experiments is plotted. Profiles for static versus static + Calp3 are significantly different from each other (*P* < 0.001) by two‐way ANOVA. (B) FRET measurements of OK cells transfected with the Raichu‐Cdc42‐ transfected OK cells were maintained under static conditions or exposed to 0.1 dyne/cm^2^ FSS for 30 min starting at 10 min (arrowhead). The calmodulin inhibitor W13 (60 *μ*mol/L) was included where indicated. Data (mean ± SEM) from three independent experiments are plotted. Profiles for all conditions are significantly different from each other (*P* < 0.001) by two‐way ANOVA. (C) Calp3 increases albumin uptake under static conditions. Data from seven experiments are plotted, and the bar shows the mean uptake for each condition. Representative images from a separate experiment in which cells were fixed and albumin uptake imaged using confocal microscopy are shown above each bar. Scale bar: 25 *μ*m. (D) W13 inhibits albumin uptake in response to FSS. Data from three experiments are plotted, and the bar shows the mean uptake for each condition. Representative images from a separate experiment in which cells were fixed and albumin uptake imaged using confocal microscopy are shown above each bar. Scale bar: 25 *μ*m.

Activation of Ca^2+^‐binding protein calmodulin (CaM) is a central mediator of downstream responses to increases in [Ca^2+^]_i_. To test whether CaM is required for the activation of Cdc42 in response to FSS, we measured Cdc42 activation by FRET upon addition of the CaM activator Calp3 (Villain et al. 2000). Addition of Calp3 to cells maintained under static conditions increased Cdc42 FRET (Fig. 3A), similar to our results using ryanodine. Moreover, Calp3 enhanced constitutive uptake of AlexaFluor 647‐albumin under static conditions (Fig. 3B). In contrast, when cells were exposed to FSS in the presence of the CaM inhibitor W13 (Wei et al. 1983), Cdc42 activation was not observed (Fig. 3C). Moreover, W13 blunted the endocytic response to FSS (Fig. 3D).

## Discussion

Studies in many cell types have focused on the potent role of flow in regulating cell functions. Aberrant signaling responses to mechanical stress are implicated in the progression of various diseases, including vascular disease, osteoporosis, and cancer (Iolascon et al. 2013; Baeyens et al. 2016; Brown et al. 2016; Tarbell and Cancel 2016). Similarly, tubular flow should be considered as a powerful regulator of proximal cell function. FSS resulting from fluid movement in renal tubules affects the organization of the cytoskeleton and the brush border, with consequences to many cellular functions including ion transport and endocytosis (Duan et al. 2008; Ferrell et al. 2012; Maggiorani et al. 2015).

**Figure 4:**
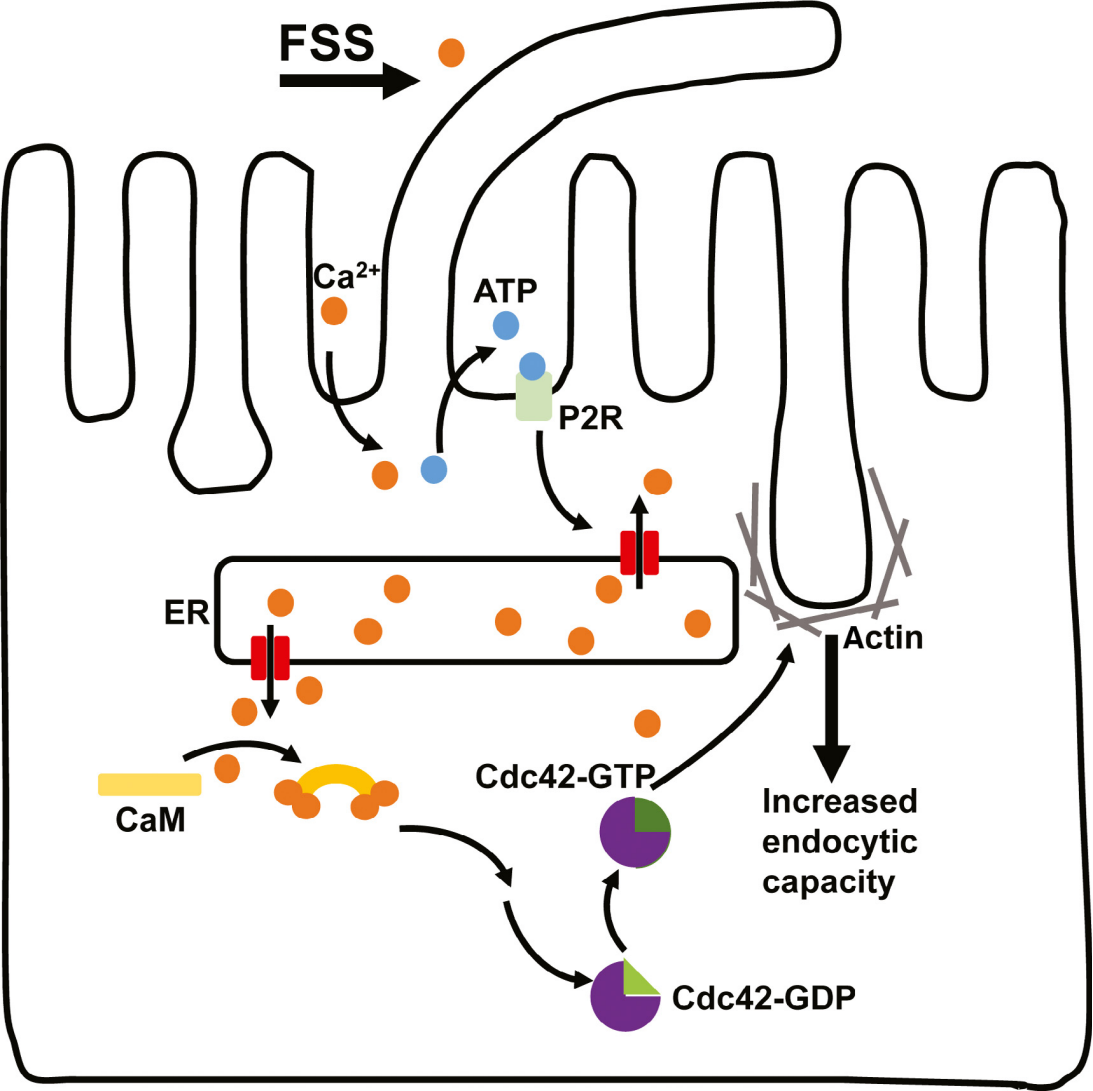
Updated model for the translation of FSS to stimulated endocytosis. Our data support a model in which exposure to FSS increases apical endocytic capacity in PT cells via a pathway that requires mechanosensation by the primary cilium. Our previous studies demonstrated that this response requires extracellular Ca^2+^ and is mediated by release of ATP and purinergic receptor (P2R) signaling that leads to release of Ca^2+^ from endoplasmic reticulum (ER) stores. Here we show that FSS‐stimulated endocytosis requires calmodulin (CaM) mediated activation of Cdc42. We hypothesize that Cdc42 modulates cytoskeletal dynamics that lead to increased endocytic capacity.

Our prior studies demonstrate that exposure of PT cells to FSS results in mobilization of [Ca^2+^]_i_ via a pathway dependent on the primary cilia. We have now investigated the downstream events triggered by the increase in [Ca^2+^]_i_ that lead to increased endocytosis. We found that activation of Cdc42 is a necessary step in the PT cell signaling cascade activated by the low levels of FSS (0.1 dyne/cm^2^) used in our studies. Mobilization of [Ca^2+^]_i_ and activation of CaM in the absence of FSS were sufficient to activate Cdc42 and enhance endocytosis. Our current model for how acute changes in FSS are coupled to endocytic capacity in PT cells is shown in Figure 4.

Cdc42 is a known master regulator of actin dynamics, and disruption of actin inhibited albumin uptake in cells maintained under static conditions as well as in response to FSS. A role for actin in apical clathrin‐dependent endocytosis has been previously demonstrated in MDCK cells (Gottlieb et al. 1993; Boulant et al. 2011). Additionally, actin is known to play a role in the uptake of large virus particles through elongated clathrin‐coated structures (Cureton et al. 2009). In this regard, it is interesting that clathrin‐coated invaginations in PT cells are highly irregular in shape (Rodman et al. 1986; Birn et al. 1993). Of note, Cdc42 is a potent activator of Toca‐1 and N‐WASP‐Arp2/3, which regulate the formation of branched actin filaments and which have previously been implicated in endocytosis (Bu et al. 2010). We hypothesize that activation of N‐WASP/Arp2/3 leads to structural remodeling of the actin cytoskeleton to enable the formation of clathrin‐coated structures.

Our data also suggest that the [Ca^2+^]_i_ response to flow varies with shear stress. We previously found that renal cell exposure to higher FSS (≥2 dyne/cm^2^) results in a rapid and transient increase in [Ca^2+^]_i._ The lower FSS used here also led to an initial spike in [Ca^2+^]_i_ but this quickly reset to a higher baseline [Ca^2+^]_i_ that remained steady for at least 10 min. This difference in Ca^2+^ responses could reflect variable sensitivity to FSS of the machinery needed to re‐equilibrate [Ca^2+^]_i_. Estimates of FSS experienced by PT cells in vivo vary widely, and cellular responses to higher levels of FSS may differ from those we observed at the lower end of the reported range. Interestingly, a recent study using a genetically encoded Ca^2+^ sensor in mice demonstrated that the effect of ATP on PT [Ca^2+^]_i_ levels in vivo is considerably longer than in vitro (Szebenyi et al. 2015). Thus, Ca^2+^ responses to physiologic stimuli may be longer lived in the PT than previously thought.

Our studies point to a critical role for primary cilia in the activation of Cdc42 by FSS, however, we cannot rule out the possibility that our method of deciliation has nonciliary effects on cell function. That said, our deciliated cells are clearly viable, have intact plasma membranes, and are able to regrow cilia. It is generally accepted that primary cilia are required for flow‐dependent mechanosensation; however, their specific role in signaling remains unknown. Flow‐sensitive Ca^2+^ responses in distal tubules of mice with impaired ciliary biogenesis are blunted, and deciliation has also been shown to impair the flow‐dependent Ca^2+^ response in MDCK cells (Liu et al. 2005; Praetorius and Leipziger 2009). However, other studies have reported cilium‐independent increases in [Ca^2+^]_i_ in response to FSS (Liu et al. 2003; Rodat‐Despoix et al. 2013). Moreover, changes in Ca^2+^ within the cilium occur subsequent to cytoplasmic changes when cells are stimulated with ATP or FSS (Su et al. 2013; Delling et al. 2016).

In summary, the studies described here deepen our understanding of how acute changes in FSS modulate endocytic capacity. Activation of Cdc42 in response to cilia‐initiated changes in [Ca^2+^]_i_ is a necessary step in this cascade, and is regulated upstream by CaM. Activation of Cdc42 may also contribute to other effects of FSS on PT function, including the modulation of tight junction dynamics (Rojas et al. 2001; Duan et al. 2008). Future studies will be aimed at identifying the downstream targets of Cdc42 in response to FSS and the mechanism by which endocytic capacity is enhanced in PT cells in culture and in vivo.

## Conflict of Interest

The authors declare that they have no conflicts of interest with the contents of this article.

## References

[phy213460-bib-0001] Baeyens, N. , C. Bandyopadhyay , B. G. Coon , S. Yun , and M. A. Schwartz . 2016 Endothelial fluid shear stress sensing in vascular health and disease. J. Clin. Invest. 126:821–828. 26928035 10.1172/JCI83083 PMC4767335

[phy213460-bib-0002] Birn, H. , E. I. Christensen , and S. Nielsen . 1993 Kinetics of endocytosis in renal proximal tubule studied with ruthenium red as membrane marker. Am. J. Physiol. 264:F239–F250. 7680532 10.1152/ajprenal.1993.264.2.F239

[phy213460-bib-0003] Boulant, S. , C. Kural , J. C. Zeeh , F. Ubelmann , and T. Kirchhausen . 2011 Actin dynamics counteract membrane tension during clathrin‐mediated endocytosis. Nat. Cell Biol. 13:1124–1131. 21841790 10.1038/ncb2307 PMC3167020

[phy213460-bib-0004] Brown, A. J. , Z. Teng , P. C. Evans , J. H. Gillard , H. Samady , and M. R. Bennett . 2016 Role of biomechanical forces in the natural history of coronary atherosclerosis. Nat. Rev. Cardiol. 13:210–220. 26822720 10.1038/nrcardio.2015.203

[phy213460-bib-0005] Bu, W. , K. B. Lim , Y. H. Yu , A. M. Chou , T. Sudhaharan , and S. Ahmed . 2010 Cdc42 interaction with N‐WASP and Toca‐1 regulates membrane tubulation, vesicle formation and vesicle motility: implications for endocytosis. PLoS ONE 5:e12153. 20730103 10.1371/journal.pone.0012153 PMC2921345

[phy213460-bib-0006] Cureton, D. K. , R. H. Massol , S. Saffarian , T. L. Kirchhausen , and S. P. Whelan . 2009 Vesicular stomatitis virus enters cells through vesicles incompletely coated with clathrin that depend upon actin for internalization. PLoS Pathog. 5:e1000394. 19390604 10.1371/journal.ppat.1000394 PMC2667253

[phy213460-bib-0007] Delling, M. , A. A. Indzhykulian , X. Liu , Y. Li , T. Xie , D. P. Corey , et al. 2016 Primary cilia are not calcium‐responsive mechanosensors. Nature 531:656–660. 27007841 10.1038/nature17426 PMC4851444

[phy213460-bib-0008] Du, Z. , Y. Duan , Q. Yan , A. M. Weinstein , S. Weinbaum , and T. Wang . 2004 Mechanosensory function of microvilli of the kidney proximal tubule. Proc. Natl Acad. Sci. USA 101:13068–13073. 15319475 10.1073/pnas.0405179101 PMC516518

[phy213460-bib-0009] Duan, Y. , N. Gotoh , Q. Yan , Z. Du , A. M. Weinstein , T. Wang , et al. 2008 Shear‐induced reorganization of renal proximal tubule cell actin cytoskeleton and apical junctional complexes. Proc. Natl Acad. Sci. USA 105:11418–11423. 18685100 10.1073/pnas.0804954105 PMC2516248

[phy213460-bib-0010] Eshbach, M. L. , and O. A. Weisz . 2017 Receptor‐mediated endocytosis in the proximal tubule. Annu. Rev. Physiol. 79:425–448. 27813828 10.1146/annurev-physiol-022516-034234 PMC5512543

[phy213460-bib-0011] Essig, M. , and G. Friedlander . 2003 Tubular shear stress and phenotype of renal proximal tubular cells. J. Am. Soc. Nephrol. 14(Suppl 1):S33–S35. 12761236 10.1097/01.asn.0000067650.43083.df

[phy213460-bib-0012] Ferrell, N. , K. B. Ricci , J. Groszek , J. T. Marmerstein , and W. H. Fissell . 2012 Albumin handling by renal tubular epithelial cells in a microfluidic bioreactor. Biotechnol. Bioeng. 109:797–803. 22012446 10.1002/bit.24339 PMC3285552

[phy213460-bib-0013] Gottlieb, T. A. , I. E. Ivanov , M. Adesnik , and D. D. Sabatini . 1993 Actin microfilaments play a critical role in endocytosis at the apical but not the basolateral surface of polarized epithelial cells. J. Cell Biol. 120:695–710. 8381123 10.1083/jcb.120.3.695 PMC2119548

[phy213460-bib-0014] Hong, L. , S. R. Kenney , G. K. Phillips , D. Simpson , C. E. Schroeder , J. Noth , et al. 2013 Characterization of a Cdc42 protein inhibitor and its use as a molecular probe. J. Biol. Chem. 288:8531–8543. 23382385 10.1074/jbc.M112.435941 PMC3605667

[phy213460-bib-0015] Iolascon, G. , G. Resmini , and U. Tarantino . 2013 Mechanobiology of bone. Aging Clin. Exp. Res. 25(Suppl 1):S3–S7. 24046028 10.1007/s40520-013-0101-2

[phy213460-bib-0016] Itoh, R. E. , K. Kurokawa , Y. Ohba , H. Yoshizaki , N. Mochizuki , and M. Matsuda . 2002 Activation of rac and cdc42 video imaged by fluorescent resonance energy transfer‐based single‐molecule probes in the membrane of living cells. Mol. Cell. Biol. 22:6582–6591. 12192056 10.1128/MCB.22.18.6582-6591.2002 PMC135619

[phy213460-bib-0017] Jang, K. J. , A. P. Mehr , G. A. Hamilton , L. A. McPartlin , S. Chung , K. Y. Suh , et al. 2013 Human kidney proximal tubule‐on‐a‐chip for drug transport and nephrotoxicity assessment. Integr. Biol. (Camb) 5:1119–1129. 23644926 10.1039/c3ib40049b

[phy213460-bib-0018] Kotsis, F. , C. Boehlke , and E. W. Kuehn . 2013 The ciliary flow sensor and polycystic kidney disease. Nephrol. Dial. Transplant. 28:518–526. 23314319 10.1093/ndt/gfs524 PMC3588856

[phy213460-bib-0019] Li, S. , B. P. Chen , N. Azuma , Y. L. Hu , S. Z. Wu , B. E. Sumpio , et al. 1999 Distinct roles for the small GTPases Cdc42 and Rho in endothelial responses to shear stress. J. Clin. Invest. 103:1141–1150. 10207166 10.1172/JCI5367 PMC408275

[phy213460-bib-0020] Liu, W. , S. Xu , C. Woda , P. Kim , S. Weinbaum , and L. M. Satlin . 2003 Effect of flow and stretch on the [Ca2 + ]i response of principal and intercalated cells in cortical collecting duct. Am. J. Physiol. Renal Physiol. 285:F998–F1012. 12837680 10.1152/ajprenal.00067.2003

[phy213460-bib-0021] Liu, W. , N. S. Murcia , Y. Duan , S. Weinbaum , B. K. Yoder , E. Schwiebert , et al. 2005 Mechanoregulation of intracellular Ca2 + concentration is attenuated in collecting duct of monocilium‐impaired orpk mice. Am. J. Physiol. Renal Physiol. 289:F978–F988. 15972389 10.1152/ajprenal.00260.2004

[phy213460-bib-0022] Liu, B. , S. Lu , S. Zheng , Z. Jiang , and Y. Wang . 2011 Two distinct phases of calcium signalling under flow. Cardiovasc. Res. 91:124–133. 21285296 10.1093/cvr/cvr033 PMC3112016

[phy213460-bib-0023] Maggiorani, D. , R. Dissard , M. Belloy , J. S. Saulnier‐Blache , A. Casemayou , L. Ducasse , et al. 2015 Shear stress‐induced alteration of epithelial organization in human renal tubular cells. PLoS ONE 10:e0131416. 26146837 10.1371/journal.pone.0131416 PMC4493045

[phy213460-bib-0024] Nakamura, T. , K. Kurokawa , E. Kiyokawa , and M. Matsuda . 2006 Analysis of the spatiotemporal activation of rho GTPases using Raichu probes. Methods Enzymol. 406:315–332. 16472667 10.1016/S0076-6879(06)06023-X

[phy213460-bib-0025] Nauli, S. M. , F. J. Alenghat , Y. Luo , E. Williams , P. Vassilev , X. Li , et al. 2003 Polycystins 1 and 2 mediate mechanosensation in the primary cilium of kidney cells. Nat. Genet. 33:129–137. 12514735 10.1038/ng1076

[phy213460-bib-0026] Praetorius, H. A. 2015 The primary cilium as sensor of fluid flow: new building blocks to the model. A review in the theme: cell signaling: proteins, pathways and mechanisms. Am. J. Physiol. Cell Physiol. 308:C198–C208. 25428884 10.1152/ajpcell.00336.2014

[phy213460-bib-0027] Praetorius, H. A. , and J. Leipziger . 2009 Released nucleotides amplify the cilium‐dependent, flow‐induced [Ca2 + ]i response in MDCK cells. Acta Physiol. (Oxf) 197:241–251. 19432587 10.1111/j.1748-1716.2009.02002.x

[phy213460-bib-0028] Raghavan, V. , and O. A. Weisz . 2015 Flow stimulated endocytosis in the proximal tubule. Curr. Opin. Nephrol. Hypertens. 24:359–365. 26050123 10.1097/MNH.0000000000000135 PMC4494861

[phy213460-bib-0029] Raghavan, V. , Y. Rbaibi , N. M. Pastor‐Soler , M. D. Carattino , and O. A. Weisz . 2014 Shear stress‐dependent regulation of apical endocytosis in renal proximal tubule cells mediated by primary cilia. Proc. Natl Acad. Sci. USA 111:8506–8511. 24912170 10.1073/pnas.1402195111 PMC4060694

[phy213460-bib-0030] Rodat‐Despoix, L. , J. Hao , M. Dandonneau , and P. Delmas . 2013 Shear stress‐induced Ca(2)(+) mobilization in MDCK cells is ATP dependent, no matter the primary cilium. Cell Calcium 53:327–337. 23528238 10.1016/j.ceca.2013.02.002

[phy213460-bib-0031] Rodman, J. S. , L. Seidman , and M. G. Farquhar . 1986 The membrane composition of coated pits, microvilli, endosomes, and lysosomes is distinctive in the rat kidney proximal tubule cell. J. Cell Biol. 102:77–87. 2867100 10.1083/jcb.102.1.77 PMC2114052

[phy213460-bib-0032] Rohatgi, R. , and D. Flores . 2010 Intratubular hydrodynamic forces influence tubulointerstitial fibrosis in the kidney. Curr. Opin. Nephrol. Hypertens. 19:65–71. 19851105 10.1097/MNH.0b013e32833327f3 PMC2887746

[phy213460-bib-0033] Rojas, R. , W. G. Ruiz , S. M. Leung , T. S. Jou , and G. Apodaca . 2001 Cdc42‐dependent modulation of tight junctions and membrane protein traffic in polarized Madin‐Darby canine kidney cells. Mol. Biol. Cell 12:2257–2274. 11514615 10.1091/mbc.12.8.2257 PMC58593

[phy213460-bib-0034] Rydholm, S. , G. Zwartz , J. M. Kowalewski , P. Kamali‐Zare , T. Frisk , and H. Brismar . 2010 Mechanical properties of primary cilia regulate the response to fluid flow. Am. J. Physiol. Renal Physiol. 298:F1096–F1102. 20089672 10.1152/ajprenal.00657.2009

[phy213460-bib-0035] Su, S. , S. C. Phua , R. DeRose , S. Chiba , K. Narita , P. N. Kalugin , et al. 2013 Genetically encoded calcium indicator illuminates calcium dynamics in primary cilia. Nat. Methods 10:1105–1107. 24056873 10.1038/nmeth.2647 PMC3860264

[phy213460-bib-0036] Surviladze, Z. , A. Waller , J. J. Strouse , C. Bologa , O. Ursu , V. Salas , et al. 2010 A potent and selective inhibitor of Cdc42 GTPase Probe reports from the NIH Molecular Libraries Program. Bethesda (MD).

[phy213460-bib-0037] Szebenyi, K. , A. Furedi , O. Kolacsek , R. Csohany , A. Prokai , K. Kis‐Petik , et al. 2015 Visualization of calcium dynamics in kidney proximal tubules. J. Am. Soc. Nephrol. 26:2731–2740. 25788535 10.1681/ASN.2014070705 PMC4625667

[phy213460-bib-0038] Tarbell, J. M. , and L. M. Cancel . 2016 The glycocalyx and its significance in human medicine. J. Intern. Med. 280:97–113. 26749537 10.1111/joim.12465

[phy213460-bib-0039] Thompson, G. , D. Owen , P. A. Chalk , and P. N. Lowe . 1998 Delineation of the Cdc42/Rac‐binding domain of p21‐activated kinase. Biochemistry 37:7885–7891. 9601050 10.1021/bi980140+

[phy213460-bib-0040] Tzima, E. , W. B. Kiosses , M. A. del Pozo , and M. A. Schwartz . 2003 Localized cdc42 activation, detected using a novel assay, mediates microtubule organizing center positioning in endothelial cells in response to fluid shear stress. J. Biol. Chem. 278:31020–31023. 12754216 10.1074/jbc.M301179200

[phy213460-bib-0041] Villain, M. , P. L. Jackson , M. K. Manion , W. J. Dong , Z. Su , G. Fassina , et al. 2000 De novo design of peptides targeted to the EF hands of calmodulin. J. Biol. Chem. 275:2676–2685. 10644729 10.1074/jbc.275.4.2676

[phy213460-bib-0042] Wei, J. W. , R. A. Hickie , and D. J. Klaassen . 1983 Inhibition of human breast cancer colony formation by anticalmodulin agents: trifluoperazine, W‐7, and W‐13. Cancer Chemother. Pharmacol. 11:86–90. 6627600 10.1007/BF00254251

[phy213460-bib-0043] Weinbaum, S. , Y. Duan , L. M. Satlin , T. Wang , and A. M. Weinstein . 2010 Mechanotransduction in the renal tubule. Am. J. Physiol. Renal Physiol. 299:F1220–F1236. 20810611 10.1152/ajprenal.00453.2010 PMC3006307

[phy213460-bib-0044] Xu, C. , B. E. Shmukler , K. Nishimura , E. Kaczmarek , S. Rossetti , P. C. Harris , et al. 2009 Attenuated, flow‐induced ATP release contributes to absence of flow‐sensitive, purinergic Cai2 + signaling in human ADPKD cyst epithelial cells. Am. J. Physiol. Renal Physiol. 296:F1464–F1476. 19244404 10.1152/ajprenal.90542.2008 PMC2692447

[phy213460-bib-0045] Yoder, B. K. 2007 Role of primary cilia in the pathogenesis of polycystic kidney disease. J. Am. Soc. Nephrol. 18:1381–1388. 17429051 10.1681/ASN.2006111215

